# Cushing's Disease: The Relevance of a Combined Dexamethasone Desmopressin Test as a Component of Postoperative Hormonal Evaluation

**DOI:** 10.1155/2015/357165

**Published:** 2015-06-22

**Authors:** Przemysław Witek, Grzegorz Zieliński, Katarzyna Szamotulska

**Affiliations:** ^1^Department of Endocrinology and Isotope Therapy, Military Institute of Medicine, Ul. Szaserów 128, 04-141 Warsaw, Poland; ^2^Department of Neurosurgery, Military Institute of Medicine, Ul. Szaserów 128, 04-141 Warsaw, Poland; ^3^Department of Epidemiology, Institute of Mother and Child, Ul. Kasprzaka 17a, 01-211 Warsaw, Poland

## Abstract

*Background*. The risk of Cushing's disease (CD) recurring may persist for years, even after initially successful surgery.* Objective*. To prospectively assess the relevance of a combined dexamethasone desmopressin test (CDDT) as a component of postoperative hormonal evaluation, including the dynamics of ACTH and cortisol concentrations.* Material and Methods*. We included 28 patients after TSS for CD. Eighteen months after surgery the standard hormonal evaluation was performed, followed by a CDDT.* Results*. Fifteen patients (53.6%) were in remission whereas in 13 subjects (46.4%) hypercortisolemia was confirmed. Positive results of CDDT were observed in 12 noncured patients (92.3%) and in one subject in remission (6.7%). Negative results were obtained in 12 patients with remission (80%) and in one noncured patient (7.7%). With 2 patients in CD remission (13.3%) the test results were inconclusive. We confirmed a high compatibility between CDDT and standard hormonal assessment results (*κ* = 0.846; *P* < 0.001). Significant differences in ACTH and cortisol levels at each CDDT time point between the two studied subgroups were shown.* Conclusions*. A negative CDDT result can be regarded as one of the factors indicative of CD remission during follow-up. Additionally, CDDT can help distinguish persistent hypercortisolemia from naturally recurring adrenal function after TSS.

## 1. Introduction

The diagnosis and treatment of Cushing's disease (CD) are among the greatest challenges in endocrinology. The diagnostic criteria of CD and the treatment principles in typical cases have been established according to a consensus of experts [1, 2]. The treatment of choice is transsphenoidal selective adenomectomy performed by an experienced neurosurgeon. However, postoperative management protocols differ across centres and countries [3–5]. Even after initially effective transsphenoidal removal of a corticotroph adenoma, the 20–30% risk of recurrence, even many years later, remains a major concern [6].

Some centres use a stimulation test with the synthetic vasopressin analog desmopressin (1-deamino-8D-arginine vasopressin, DDAVP) as a means of differentiating CD from both pseudo-Cushing states and the ectopic ACTH-dependent Cushing's syndrome. Some authors suggested the use of this test during postoperative follow-up, with a persistent response to desmopressin (positive test result) indicative of persistent Cushing's disease or a higher risk of recurrence [7–10]. Castinetti et al. suggested the use of a combined 1 mg dexamethasone suppression and desmopressin stimulation test in evaluating the risk of hypercortisolemia recurrence [9]. The addition of dexamethasone was to increase specificity of the test by suppressing the normal corticotroph cells of the pituitary while stimulating only residual tumor cells. However, a desmopressin test is not typically used in routine postoperative diagnostics. In accordance with the Endocrine Society recommendations, it should only be performed as part of research studies [1, 2].

The aim of this study was to conduct a prospective evaluation of the role and usefulness of the combined dexamethasone desmopressin test in postoperative assessments of transsphenoidal surgery effectiveness in CD patients in a single pituitary center.

## 2. Material and Methods

### 2.1. Patient Population

The study population comprised 28 consecutive patients with Cushing's disease (22 women and 6 men; F : M ratio: 3.7 : 1) hospitalized and operated in the Military Institute of Medicine in Warsaw between 2010 and 2012. The mean age was 36.1 ± 13.5 years (16.8–57.6). After their confirmed diagnosis of Cushing's disease the patients were operated on by the same neurosurgeon using the same surgical protocol.

All patients were informed about the aims and methods of the study and they signed the informed consent. The study protocol was approved by the local ethics committee.

### 2.2. Preoperative Endocrine Evaluation

All patients underwent a standard clinical evaluation. The diagnosis of ACTH-dependent Cushing's syndrome (CS) was made based on clinical signs and standard hormonal criteria: increased urinary free cortisol (UFC), increased morning serum cortisol level at 8:00, loss of cortisol circadian rhythm (the serum cortisol level above 7.5 *μ*g/dL in the late-night hours (22:00–00:00)) and increased or detectable level of plasma ACTH at 8:00, and the failure of serum cortisol to suppress to less than or equal to 1.8 *μ*g/dL during the low dose dexamethasone suppression test (LDDST; 0.5 mg q.i.d. for 48 hours). The pituitary etiology of CS was confirmed based on serum cortisol or UFC suppression greater than 50% with the high dose dexamethasone suppression test (HDDST; 2 mg q.i.d. for 48 h) and positive pituitary MRI. In the case of inconclusive hormonal assessments and pituitary imaging, the diagnosis of Cushing's disease was confirmed by a positive result of a stimulation test with intravenous CRH injection (100 *μ*g) and/or results of inferior petrosal sinus sampling (IPSS).

### 2.3. Preoperative MRI

Prior to neurosurgical treatment, all patients underwent high-resolution magnetic resonance imaging of the pituitary-hypothalamic region (*SIEMENS Symphony 2004*; 1.5 Tesla), performed before and after intravenous injection of gadolinium (Gd-DTPA). The presence, size, and position of any focal lesions were recorded. A hypodense lesion visualized after contrast administration was considered to be indicative of pituitary adenoma. Microadenoma was defined as a pituitary tumor with a diameter of less than 1 cm, whereas macroadenoma was defined as a pituitary tumor with at least one diameter of more than 1 cm.

### 2.4. Surgical Procedure

A microsurgical transseptal transsphenoidal approach was used for resection of ACTH-secreting pituitary adenomas. The pituitary gland was carefully examined, regardless of MRI findings. Selective adenomectomy was performed in all cases of MRI-visualized pituitary adenomas. Where MRI findings were inconclusive or no tumor was evident, a series of vertical and horizontal incisions of the pituitary gland were performed with all tissue deemed abnormal removed and submitted for pathological examination.

### 2.5. Histopathological and Immunohistochemical Assessment

Surgical specimens were collected for histopathological analysis (standard hematoxylin and eosin staining) and immunohistochemical staining for pituitary hormones (kits with polyclonal antibodies;* Dako*,* Denmark*). The result of a histopathological assessment was considered to be* positive* if the presence of corticotroph adenoma as well as positive ACTH-staining was confirmed in histological and immunohistochemical examinations. The result was treated as* negative* if there was no histopathological evidence of corticotroph adenoma and ACTH-staining was negative.

### 2.6. Postoperative Hormonal Evaluation and Criteria of Cure

Blood samples for serum cortisol measurements were collected from all patients at 6:00 on the first postoperative day. Glucocorticoids were not administered in the perioperative or in the early postoperative period. Hydrocortisone replacement therapy was started after biochemical confirmation of hypocortisolemia or the development of clinical manifestations of adrenal insufficiency. The standard dose of hydrocortisone (20 mg in the morning and 10 mg at 15:00) was introduced when necessary and continued until the next hormonal evaluation.

Following the surgical procedure, all patients were subjected to further postoperative evaluations. Subsequent reassessments were performed at 6 weeks and at 3, 6, 12, 18, and 24 months after surgery and once yearly thereafter. Patients on hydrocortisone replacement therapy had their cortisol measurements taken 48 hours after the last administered dose. We used the following criteria for sustained remission: biochemical evidence of eucortisolemia (morning serum cortisol in the 5–25 *μ*g/dL range), preserved circadian rhythm of serum cortisol (the late-night serum cortisol level ≤7.5 *μ*g/dL), and the ability of serum cortisol to suppress to less than or equal to 1.8 *μ*g/dL after a 1 mg overnight dexamethasone suppression test (ODST).

### 2.7. Combined Dexamethasone Desmopressin Testing

The combined dexamethasone desmopressin test (CDDT) was conducted in two stages. After the informed consent had been signed, each patient received 1 mg of oral dexamethasone at midnight. In patients who had received hydrocortisone replacement therapy it was discontinued 48 hours prior to the test. A 10 *μ*g of desmopressin—Minirin (Ferring GmbH, Germany)—was administered as a slow, intravenous bolus 8 hours after dexamethasone administration. Blood samples for testing ACTH and cortisol levels were collected before and 15, 30, 60, 90, and 120 minutes after desmopressin administration.

A positive test result, which indicates persistent, improper corticotroph secretory response to desmopressin, requires plasma ACTH increase by at least 30% and serum cortisol by at least 20% compared to baseline [7]. The baseline values correspond to plasma ACTH and serum cortisol following ODST. In addition, due to low ACTH and cortisol levels in patients after successful TSS, we adopted an additional criterion necessary for interpreting the test result as positive. This was an absolute increase in plasma ACTH by ≥10 pg/mL and serum cortisol by ≥2 *μ*g/dL. An increase only in ACTH levels without a parallel increase in cortisol levels was interpreted as inconclusive.

### 2.8. Hormone Assay

Chemiluminescent immunometric assays (IMMULITE 2000;* Siemens*, UK) were used to measure serum cortisol. Method sensitivity was 0.2 *μ*g/dL (5.5 nmol/L) and the reference range 5.0–25.0 *μ*g/dL. The quantification threshold for serum cortisol levels, using the analyser, was the measured value of 1.0 *μ*g/dL. Thus, for the purpose of further calculations it was assumed that in these cases the postoperative cortisol was 1.0. Plasma ACTH levels were measured using a specific two-step radioimmunometric assay (IRMA;* coated tube* technique; Brahms, Germany). Method sensitivity was 1.2 pg/mL (reference range: 10–60 pg/mL).

### 2.9. Statistical Analysis

The statistical analysis employed methods of descriptive statistics (mean, median, standard deviation, and proportion). The hypotheses concerning the relationship between two categorical variables were verified using the exact chi-square test (Fisher's exact test). The significance of the differences between average values of continuous variables in two groups was analyzed by means of the Mann-Whitney *U* test for small samples. Verification of hypotheses concerning comparisons of the analyzed parameters in two time points was conducted using the Wilcoxon test for small samples.

The dynamics of analyzed tests results was conducted by calculating areas under the curve (AUCs) using the trapezoid rule. The significance of differences between AUCs was checked using the Mann-Whitney *U* test. In order to present average values and dispersion of AUCs, mean and standard errors of logarithmically transformed values were used. The transformation was performed to obtain the normal distribution of AUCs.

The level of significance was set at *P* < 0.05. The calculations were made using the commercially available statistical software package SPSS v.18.0.

## 3. Results

Thirty-six consecutive patients after transsphenoidal surgery for CD participating in the structured postoperative follow-up program were offered an opportunity to undergo a CDDT 18 months after TSS. A total of 28 patients (77.8%) consented, including 15 (out of a total of 23) patients (65.2%) who were in remission of hypercortisolemia at 18 months following surgery and all of the 13 patients with persistent hypercortisolemia. Out of the 8 patients considered to be in remission who did not undergo the test there were 3 pregnant or breastfeeding females and 5 patients who did not provide their informed consent.

### 3.1. Pre- and Perioperative Characteristics

Detailed demographic data, preoperative characteristics of the study group, preoperative and early postoperative hormone level results, and immunohistochemical findings are presented in Table 1.

### 3.2. Results of Postoperative Hormone Assessments

Eighteen months after TSS, the mean serum cortisol levels at 8:00 in the entire study group were 14.8 ± 7.7 *μ*g/dL (median: 14; range: 5.3–32.4 *μ*g/dL). Normal circadian rhythms of serum cortisol were found in 17 patients (61%). Mean serum cortisol levels in an overnight dexamethasone suppression test (ODST) in the whole group were 4.08 ± 4.23 *μ*g/dL (median: 1.7 *μ*g/dL; range: 1.0–17.8 *μ*g/dL). According to the adopted criteria, 15 patients (53.6%) with serum cortisol levels at 8:00 within the referral range, normal circadian rhythm, and post-ODST cortisol levels below 1.8 *μ*g/dL were found to be in remission of hypercortisolemia. The remaining 13 patients (46.4%) were considered to have persistent hypercortisolemia. In these two patient subgroups mean serum cortisol levels at 8:00 were 10.1 ± 3.5 *μ*g/dL (median: 10.2; range: 5.3–16.4 *μ*g/dL) and 20.3 ± 7.6 *μ*g/dL (median: 16.8; range: 11.6–33.1 *μ*g/dL), respectively; *P* < 0.001. Mean cortisol levels after ODST were 1.17 ± 0.24 *μ*g/dL (median: 1.0; range: 1–1.7 *μ*g/dL) and 7.46 ± 4.14 *μ*g/dL (median: 7.7; range: 3.2–17.8 *μ*g/dL), respectively; *P* < 0.001. Mean nadir serum cortisol levels on the 1st or 2nd postoperative day (determined retrospectively) in the remission and uncured subgroups were 1.5 *μ*g/dL ± 0.5 (median: 1.6; range: 1–2.5 *μ*g/dL) and 14.0 ± 11.4 (median: 8.7; range: 4.1–37.0), respectively; *P* < 0.001.

### 3.3. Results of CDDT

At first, CDDT results were analyzed qualitatively. According to the predetermined criteria the test results were positive in 13 out of 28 patients (46.4%), negative in 13 patients (46.4%), and inconclusive in 2 patients (7.2%). A comparison of the CDDT results with typically performed hormonal assessments at month 18 following TSS revealed positive test results in 12 out of 13 uncured patients (92.3%) and in 1 out of 15 patients (6.7%) considered to be in remission after 18 months. Negative test results were observed in 12 out of 15 patients (80%) in remission of Cushing's disease and in 1 out of 13 uncured patients (7.7%). CDDT results proved to be highly consistent with surgical efficacy assessments based on the conventional hormonal criteria presented above (*κ* = 0.846; *P* < 0.001). The test was inconclusive in 2 out of 15 patients (13.3%) who met hormonal criteria for remission of hypercortisolemia. In both cases only plasma ACTH elevation was observed, without a concomitant increase in serum cortisol levels.

Test results were analyzed separately in the remission subgroup and in the uncured CD subgroup. Analysis findings are presented in Table 2. There were significant differences in terms of cortisol levels between the remission and uncured subgroups (*P* < 0.001) at all evaluated time points. Moreover, the two subgroups differed significantly in terms of plasma ACTH levels in respective, consecutive test time points (*P* < 0.001).

Subsequently, the remission and uncured groups were compared in terms of absolute (Δ) and relative (Δ%) changes in cortisol levels at different test time points versus baseline values. A similar comparison was then conducted for absolute (Δ) and relative (Δ%) changes in ACTH levels. The group in remission of hypercortisolemia and the uncured group differed significantly in terms of cortisol levels at every time point. Similar findings were shown in terms of ACTH levels at every time point except for the relative change in minute 15 of the test. Detailed findings have been presented in Tables 3 and 4.

Six patients (46.2%) from the uncured subgroup (*n* = 13) reached maximum ACTH levels at minute 15, while the remaining 7 patients (53.8%) reached maximum ACTH levels at minute 30. The maximum increase in cortisol levels following the CDDT was observed in 1 patient (7.7%) at minute 15, in 2 patients (15.2%) at minute 30, in 7 patients (53.9%) at minute 60, in 2 patients (15.3%) at minute 90, and in the case of 1 patient (7.7%) at minute 120.

Two patients who met the criteria for CD remission and in whom test results were inconclusive achieved the maximum plasma ACTH levels at 15 minutes. One of these patients achieved an increase in plasma ACTH levels from 15 pg/mL at the starting point to 25 pg/mL at 15 minutes and the other patient from 3 pg/mL to 16 pg/mL, respectively. Serum cortisol levels in both patients were below 1.0 *μ*g/dL and did not increase throughout the test.

**Figure 1:**
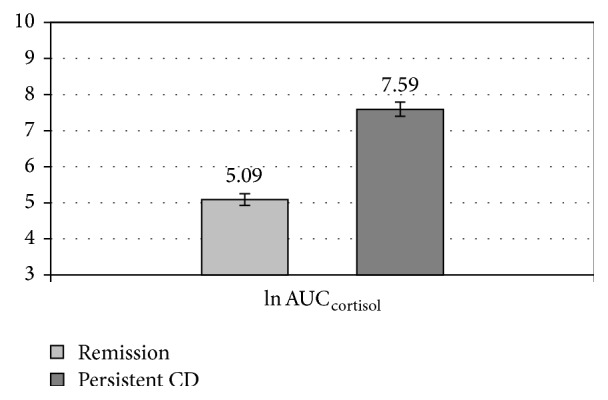
The mean individual log-transformed cortisol area under the curve (AUC) obtained in the CDDT in CD remission and uncured patients (*P* < 0.001), with standard errors of the mean (SEM).

**Figure 2:**
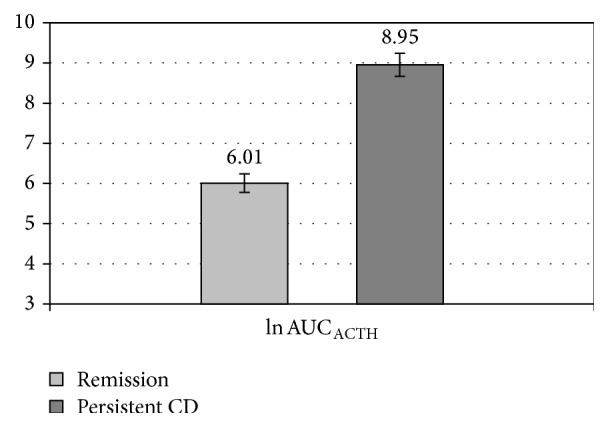
The mean individual log-transformed ACTH area under the curve (AUC) obtained in the CDDT in Cushing's disease remission and uncured patients (*P* < 0.001), with standard errors of the mean (SEM).

We also compared the two subgroups in terms of areas under the curve (AUCs) for the tested ACTH and cortisol levels. The AUC_cortisol_ values in the remission and uncured subgroups were as follows: mean 218.46 ± 284 *μ*g/dL, median 125.4, and range 120–1,230.45 *μ*g/dL and mean 2,340.81 ± 1,056.6 *μ*g/dL, median 2,591.25, and range 335.33–4,065.75 *μ*g/dL, respectively (*P* < 0.001). The AUC_ACTH_ values in the remission and uncured groups were as follows: mean 609.95 ± 642.52 *μ*g/dL, median 427.5, and range 124.5–2,227.5 pg/mL and mean 13,371.92 ± 18,467.9 pg/mL, median 8,190, and range 1,875–71,805 *μ*g/dL, respectively (*P* < 0.001). Following a logarithmic transformation, the individual log AUC values for ACTH and cortisol were compared and presented as mean and standard error of the mean (SEM). The mean individual log-transformed AUC_cortisol_ values in the CD remission and uncured subgroups were as follows: 5,09 ± 0,62 (SEM 0.16; median 4.83; range 4.79–7.12) and 7.59 ± 0.71 (SEM 0.2; median 7.86; range 5.82–8.31), respectively (*P* < 0.001). The mean individual log-transformed AUC_ACTH_ values in the remission and uncured groups were as follows: 6.01 ± 0.9 (SEM 0.23; median 6.06; range 4.82–7.71) and 8.95 ± 1.04 (SEM 0.29; median 9.01; range 7.54–11.8), respectively (*P* < 0.001) (see Figures 1 and 2).

Our results suggest that Cushing's disease remission group and persistent CD group differ significantly in terms of combined dexamethasone suppression and desmopressin stimulation test results. The relative and absolute changes in cortisol and ACTH levels were evaluated in both subgroups at every test time point. The CDDT results are highly consistent with the traditional hormonal assessments at month 18 following transsphenoidal pituitary surgery.

## 4. Discussion

The desmopressin-induced ACTH and cortisol stimulation test has been used in CD diagnostics since the mid-90s [11–13], in several European and American centres. Its use is based on the confirmed presence of V3 vasopressin receptors expressed on the cells of the majority of pituitary corticotroph tumors [12]. Desmopressin (DDAVP) is a vasopressin analogue that lacks an amine group at position 1 and that has D-arginine at position 8, in place of the physiologically occurring L-isomer. These modifications lead to a prolonged biological activity of the analogue and its significantly larger selectivity towards the V3 receptor, in comparison with the original molecule. This translates into the elimination of the vasoconstriction and hypertensive effect, which definitely increases the safety of DDAVP use, compared with vasopressin. Initially, the desmopressin test was used only in the differential diagnostics of ACTH-dependent Cushing's syndrome [14, 15]. The test was not explicitly regarded by endocrine societies a management standard, although it gained popularity, especially in Italian and French centres. Some literature data suggest that the desmopressin test is characterized by better diagnostic accuracy in differentiating mild-to-moderate cases of Cushing's disease from the so-called pseudo-Cushing's states [13–15] in comparison to a corticoliberin test performed after a low dose dexamethasone suppression test (4 × 0.5 mg for 2 days), recommended by the consensus of 2008 [1]. Hence, it seems that diagnosing Cushing's disease with moderate hypercortisolemia will constitute, in the near future, the fundamental area for diagnostic use of the test, more so as the European recommendations and commentaries indicate purposefulness of conducting scientific research towards this direction. Simultaneously, after years of gathering experience in the diagnostics of Cushing's syndrome, the DDAVP stimulation test started to be analyzed in terms of its use in the assessment of the efficacy of surgical treatment of corticotroph pituitary adenomas [7, 8].

In the dissertation published in 2009, Losa et al. demonstrated that persistent postoperative ACTH and cortisol response to DDAVP in the first week after surgical treatment increases the probability of CD recurrence. A postoperative response to desmopressin was to suggest a persistent presence of tumor cells, which are characterized by the autonomy of ACTH secretion. In such cases, desmopressin stimulation would lead to a significant increase of both ACTH and cortisol secretion. However, the main objection raised against this test is the possibility of V3 receptors being present on normal corticotroph cells, although the frequency of this phenomenon has not been explicitly determined [7]. Yet, the two indisputable advantages of this test are its wide availability and a considerably lower cost compared to those of the CRH test [2, 10].

Our study attempted to assess the frequency and clinical significance of a positive desmopressin test result, performed after surgical treatment of corticotroph pituitary adenoma. We also compared the obtained results of this test with treatment efficacy assessment results (conducted on the basis of the previously discussed remission criteria) performed 18 months after TSS. However, in order to eliminate the possibility of normal corticotroph cell stimulation by desmopressin, a modified version of this test was used, which consisted in administering a DDAVP dose 8 hours after the previous administration of 1 mg of dexamethasone. This leads to the suppression of normal pituitary corticotroph cell functions by a “low" dose of dexamethasone. At that point, only autonomous cells undergo stimulation, if they are still present in the pituitary gland or the parasellar structures [9]. In our study, the test was performed not immediately after surgery, as was the case in studies by Losa et al. [7], but approximately 18 months after surgical treatment. This is because it seems that directly after surgery one can assess the functional state of the pituitary-adrenal axis on the basis of serum cortisol or urinary free cortisol levels in a 24-hour urine collection. A confirmation of secondary adrenal cortex deficiency conducted in this manner is sufficient to regard a given patient as being in remission of CD. Within the period of 18 months from surgery, patients remaining in remission gradually regain adrenal cortex function and their serum cortisol levels increase. According to our unpublished observations, over 80% of patients in remission regain their adrenal cortex function one and a half years after the operation, which makes it possible to decrease and finally discontinue their hydrocortisone replacement therapy. In the group presented here only 3 out of 15 patients in remission (20%) continued to require hydrocortisone replacement therapy (10 mg dose in all 3 cases). This is precisely at this crucial time point when the results of both the 1 mg dexamethasone test (which precedes DDAVP administration) and desmopressin stimulation test provide valuable information that helps distinguish, with a high probability, the returning of normal adrenal function from an onset of the recurrence of hypercortisolemia. In the former case, one should expect a decrease in serum cortisol levels to the value of <1.8 *μ*g/dL and a negative result of the desmopressin test and, in the latter one, cortisol levels of ≥1.8 *μ*g/dL and a positive desmopressin test result. These two clinical situations cannot be initially differentiated solely on the basis of routine serum cortisol levels or in a 24-hour urine collection, which in both cases may be within the relevant laboratory reference ranges.

Thus, it seems that a positive ACTH and cortisol response to desmopressin after the period of 1.5 years from surgical treatment may be treated as an unfavorable prognostic feature. Such results should lead to a particularly careful and prolonged postoperative follow-up, especially in light of clinical observations indicating cases of late CD recurrence, even after 10 years from the initially effective surgery [3, 6]. The important role of this test is also confirmed by the high level of conformity between the result of a CDDT and the assessment based on the established criteria of remission that include achievement of normal cortisol levels with maintained circadian rhythm and normal suppression in the 1 mg dexamethasone test.

The demonstrated conformity between the negative result of the CDDT and subnormal serum cortisol levels on day 1 or 2 after TSS for CD is particularly noteworthy. Thus, it seems that a negative test result may be an additional factor indicating CD remission.

Our results indicate that maximum plasma ACTH concentrations were achieved at the 30-minute time point of the test or earlier, and maximum cortisol concentrations in over 90% of patients were achieved within 90 minutes after DDAVP administration. Thus, it seems that for clinical purposes ACTH and cortisol levels may be measured for a shorter period of time (i.e., for up to 90 minutes) than it has been recommended by other authors, namely, for 120–180 minutes [7, 9].

The assessment of inconclusive test results, such as those which were achieved in the case of 2 patients, still remains an open question requiring further investigations (including those conducted in a larger group of patients with CD). Both of our patients with inconclusive results demonstrated a fairly significant relative increase (Δ%) in ACTH, with basically low absolute values of this hormone as well as a lack of cortisol response to DDAVP stimulation. Both of these patients were young women, aged under 25 at the onset of CD. One of them developed recurrence of hypercortisolemia after 5 years of follow-up, which required another surgery (currently the patient is in early remission 6 months after surgery). The elevated plasma ACTH levels observed in this woman during the test preceded both the clinical manifestations of hypercortisolemia and a deterioration in terms of traditional hormonal parameters. The other young woman continues her follow-up without evidence of disease recurrence. The answer to the question of how often such patients develop a recurrence of hypercortisolemia will definitely require a long-lasting follow-up and a larger sample size.

## 5. Conclusion

In conclusion, it should be stated that the presented ACTH and cortisol stimulation test after prior suppression of physiological secretion with a low dose of dexamethasone may facilitate an earlier diagnosis of a possible recurrence of hypercortisolemia. In diagnostically difficult cases the CDDT can help determine a plan of further treatment [10]. Thus, the test may be beneficial in diagnostically difficult cases of CD, particularly as part of postoperative assessments. In a broader sense this is a reminder of the fact that dynamic testing in CD constitutes a valuable and very often necessary supplement to routine hormonal assessments performed in basic conditions.

## 6. Study Strengths and Limitations

Like in other studies on CD, the main limitation of our work was a small sample size. Nonetheless, the low prevalence of CD should not discourage anyone from collecting clinical data, especially on procedures that raise doubts and have not yet become part of standard diagnostic management. Thus, in accordance with the Endocrine Society Clinical Practice Guideline and European Commentaries we evaluated prospectively a homogeneous group of patients from a single pituitary centre in order to collect more data on the clinical usefulness of the desmopressin test (in the coupled desmopressin-dexamethasone version) in postoperative follow-up of patients after surgical treatment of CD. Further limitation to this study was a relatively short follow-up period, due in part to the prospective character of this study. Also the presented version of the test has been used for a relatively short time, not allowing us to fully determine how to establish the positive and negative test result values. Perhaps this should be based on only one of the evaluated parameters—either ACTH or cortisol. The advantage of ACTH is produced by the same cells that undergo stimulation with desmopressin. Conversely, the advantage of cortisol is greater stability and higher repetition of results.

## Figures and Tables

**Table 1:** Basic pre- and postoperative characteristics of the study group.

Age^1^ (years)	36.1 ± 13.5
Males, *n* (%)	6 (21.4%)
BMI^1^ (kg/m^2^)	29.6 ± 5.8
Preoperative plasma ACTH^2^ (pg/mL)	86.0 (62.0–137.0)
Preoperative serum cortisol^1^ (*µ*g/dL)	26.7 ± 6.9
Serum cortisol after HDDST^2^ (*µ*g/dL)	3.9 (2.2–10.0)
Initial (1st) TSS, *n* (%)	21 (75.0%)
Tumor size, *n* (%)	
Microadenoma	23 (82.1%)
Macroadenoma	5 (17.9%)
Immunohistochemical confirmation of corticotroph tumor, *n* (%)	21 (75%)
Nadir serum cortisol^2^ on 1st or 2nd postoperative day (*µ*g/dL)	2.3 (1.2–8.2)

^1^Mean ± SD, ^2^median (interquartile range).

**Table 2:** Comparison of ACTH (pg/mL) and cortisol levels (*μ*g/dL) in the remission and uncured subgroups following CDDT.

	Remission (*n* = 15)			Persistent CD (*n* = 13)			*P* value
	Mean ± SD	Median	Range	Mean ± SD	Median	Range	
Cortisol (*μ*g/dL)							
0′	1.27 ± 0.57	1.0	1.0–2.9	7.72 ± 6.37	4.8	2.4–25.7	<0.001
15′	2.08 ± 3.32	1.0	1.0–14.0	16.43 ± 7.80	16.1	3.1–29.6	<0.001
30′	2.15 ± 3.52	1.1	1.0–14.8	20.61 ± 8.72	22.9	3.0–32.7	<0.001
60′	1.86 ± 2.49	1.0	1.0–10.7	22.22 ± 9.96	24.7	2.9–37.8	<0.001
90′	1.68 ± 1.80	1.0	1.0–8.0	20.86 ± 10.16	22.3	2.5–35.2	<0.001
120′	1.55 ± 1.55	1.0	1.0–7.0	18.69 ± 10.25	18.4	2.7–36.7	<0.001
ACTH (pg/mL)							
0′	3.54 ± 3.73	3.0	1.0–15.0	34.31 ± 29.54	19.0	9.0–96.0	<0.001
15′	8.56 ± 9.86	5.0	2.0–36.0	185.62 ± 297.56	91.0	20.0–1135.0	<0.001
30′	6.73 ± 7.34	4.0	1.0–25.0	162.46 ± 232.92	95.0	22.0–900.0	<0.001
60′	4.70 ± 5.28	3.0	0.6–19.0	101.15 ± 128.60	73.0	16.0–502.0	<0.001
90′	3.77 ± 4.01	2.0	0.4–16.0	85.00 ± 115.38	50.0	11.0–441.0	<0.001
120′	3.29 ± 3.20	2.0	1.0–13.0	72.69 ± 104.18	34.0	7.0–395.0	<0.001

**Table 3:** Absolute (Δ) changes in cortisol and ACTH levels at consecutive time points compared to baseline, stratified by remission status.

	Remission (*n* = 15)			Persistent CD (*n* = 13)			*P* value
	Mean ± SD	Median	Range	Mean ± SD	Median	Range	
Cortisol (*μ*g/dL)							
15′	0.81 ± 2.86	0.0	−0.1–11.1	8.71 ± 6.20	9.8	0.2–20.3	<0.001
30′	0.89 ± 3.06	0.1	−0.2–11.9	12.90 ± 7.10	13.8	0.3–22.3	<0.001
60′	0.60 ± 2.01	0.0	−0.2–7.8	14.50 ± 7.80	14.0	0.3–24.8	<0.001
90′	0.41 ± 1.32	0.0	−0.2–5.1	13.14 ± 8.55	13.7	−0.1–28.7	<0.001
120′	0.28 ± 1.07	0.0	−0.1–4.1	10.97 ± 8.77	9.2	−0.8–32.6	<0.001
ACTH (pg/mL)							
15′	5.02 ± 7.17	2.0	0.0–27.0	151.31 ± 292.89	55.0	1.0–1093.0	<0.001
30′	3.19 ± 4.42	1.0	−0.5–16.0	128.15 ± 227.38	56.0	3.0–858.0	<0.001
60′	1.16 ± 2.08	0.5	−1.0–5.0	66.85 ± 120.70	33.0	1.0–460.0	<0.001
90′	0.23 ± 1.41	0.0	−2.0–4.0	50.69 ± 106.12	25.0	2.0–399.0	<0.001
120′	−0.25 ± 1.51	0.0	−3.0–3.0	38.39 ± 96.01	9.0	−2.0–353.0	0.002

**Table 4:** Relative (Δ%) changes in cortisol and ACTH levels at consecutive time points versus baseline, stratified by remission status.

	Remission (*n* = 15)			Persistent CD (*n* = 13)			*P* value
	Mean ± SD	Median	Range	Mean ± SD	Median	Range	
Cortisol (*μ*g/dL)							
15′	32.0 ± 99.6	0.0	−2.2–389.5	185.0 ± 180.8	119.1	1.9–514.8	<0.001
30′	35.0 ± 106.6	7.9	−16.7–417.5	265.6 ± 253.7	175.2	9.7–854.9	<0.001
60′	23.3 ± 70.6	0.0	−14.2–274.1	290.3 ± 274.6	191.3	11.0–912.3	<0.001
90′	17.9 ± 47.3	0.0	−15.8–179.7	262.5 ± 261.8	151.9	−5.3–723.8	<0.001
120′	9.7 ± 37.6	0.0	−10.8–144.8	223.3 ± 249.9	124.0	−17.4–788.6	<0.001
ACTH (pg/mL)							
15′	143.4 ± 129.9	100.0	0.0–433.3	539.1 ± 882.0	177.8	5.3–2602.4	0.121
30′	91.0 ± 103.7	46.7	−25.0–300.0	461.7 ± 673.2	157.9	15.8–2042.9	0.024
60′	27.5 ± 61.2	26.7	−50.0–166.7	231.8 ± 317.0	91.7	5.3–1095.2	0.004
90′	11.4 ± 54.9	0.0	−63.6–133.3	156.1 ± 256.7	53.1	10.5–950.0	0.004
120′	1.5 ± 42.6	0.0	−50.0–100.0	119.0 ± 241.7	10.5	−22.2–840.5	0.011
